# Early Peripheral Blood *WT1* Expression Predicts Relapse After Allogeneic Hematopoietic Stem Cell Transplantation in Acute Myeloid Leukemia

**DOI:** 10.3390/ijms27104367

**Published:** 2026-05-14

**Authors:** Viktor Blaslov, Margareta Radic Antolic, Ivana Horvat, Tamara Vasilj, Zeljko Prka, Antonija Miljak, Davor Galusic, Lucana Vicelic Cutura, Marija Petric, Alen Ostojic, Pavle Roncevic, Iva Ivanko, Ivan Krecak, Goran Rincic, Lana Desnica, Ranka Serventi-Seiwerth, Ante Vulic, Mirta Mikulic, Nadira Durakovic, Radovan Vrhovac, Zinaida Peric

**Affiliations:** 1 University Hospital of Split, Spinciceva 1, 21000 Split, Croatia; 2 University Hospital Centre Zagreb, 10000 Zagreb, Croatia; 3 Clinical Hospital Dubrava, 10000 Zagreb, Croatia; 4 School of Medicine, University of Split, 21000 Split, Croatia; 5 University Hospital Centre Sisters of Mercy Zagreb, 10000 Zagreb, Croatia; 6 General Hospital Sibenik, 22000 Sibenik, Croatia; 7 School of Medicine, University of Rijeka, 51000 Rijeka, Croatia; 8 School of Medicine, University of Zagreb, 10000 Zagreb, Croatia; 9 Clinical Hospital Centre Rijeka, 51000 Rijeka, Croatia

**Keywords:** acute myeloid leukemia, *WT1*, measurable residual disease, allogeneic hematopoietic stem cell transplantation, peripheral blood, relapse, prognostic biomarker

## Abstract

Relapse remains the leading cause of treatment failure after allogeneic hematopoietic stem cell transplantation (allo-HSCT) in acute myeloid leukemia (AML). Early identification of patients at increased post-transplant relapse risk is essential to enable intensified surveillance and pre-emptive therapeutic strategies. Wilms’ tumor 1 (*WT1*) is overexpressed in most AML cases and represents a broadly applicable molecular marker; however, its utility as a peripheral blood (PB) measurable residual disease (MRD) marker after allo-HSCT remains incompletely defined. In this prospective multicenter cohort study, 43 adults with AML in complete remission underwent allo-HSCT between 2021 and 2023. *WT1* expression in PB was quantified using standardized real-time quantitative PCR before transplantation (WT1_pre) and at day +30 (WT1_30). Receiver operating characteristic analysis identified an optimal threshold for relapse prediction. A *WT1* cutoff of ≥3 copies/10^4^ *ABL* discriminated relapse risk. WT1_30 demonstrated strong prognostic performance (AUC 0.79; *p* = 0.005), whereas WT1_pre showed more modest predictive value (AUC 0.69; *p* = 0.037). Patients with WT1_30 ≥ 3 had inferior 12-month progression-free survival compared with those with WT1_30 < 3 (52.9% vs. 90.9%, *p* = 0.0059) and a higher 12-month cumulative incidence of relapse (31% vs. 9%, *p* = 0.054). WT1_pre ≥ 3 was also associated with inferior progression-free and overall survival (both *p* = 0.0008). Relapsed patients had significantly higher WT1_30 levels than non-relapsed patients (median 5.0 vs. 2.0 copies/10^4^ ABL; *p* = 0.018). Peripheral blood *WT1* expression, particularly at day +30, is associated with an increased relapse risk after allo-HSCT in AML and may support early post-transplant risk stratification. The identified cutoff should be considered exploratory and requires validation in larger independent cohorts.

## 1. Introduction

Acute myeloid leukemia (AML) is a clonal disorder of hematopoietic tissue characterized by the uncontrolled proliferation of myeloid blasts and subsequent bone marrow failure. It is a relatively rare malignancy, with an incidence of approximately 3–5 cases per 100,000 individuals annually in Europe. Treatment strategies have evolved significantly, with intensive chemotherapy remaining the mainstay for fit patients, while hypomethylating agents and venetoclax-based regimens are increasingly used for those unfit for intensive therapy [1,2]. Despite risk-adapted treatment and the introduction of allogeneic hematopoietic stem cell transplantation (allo-HSCT), relapse remains the primary cause of treatment failure. Current prognostic classification relies on cytogenetic and molecular markers, as defined by the European LeukemiaNet (ELN), yet these markers are identifiable in only approximately 40% of AML patients [3]. This leaves the majority in an intermediate-risk category, where predicting outcome and guiding post-remission therapy is challenging. Morphological complete remission, defined by less than 5% blasts in the bone marrow, is no longer considered sufficient for response assessment, as measurable residual disease (MRD) below this threshold can still drive relapse [4]. Accordingly, the detection of MRD is becoming a critical prognostic and therapeutic tool. MRD can be monitored by multiparametric flow cytometry or molecular techniques such as real-time quantitative polymerase chain reaction (RQ-PCR), each with distinct advantages and limitations.

Among molecular MRD targets, the *Wilms’ Tumor 1 (WT1)* gene has emerged as one of the most promising. *WT1* encodes a zinc-finger transcription factor that is overexpressed in 85–90% of AML cases, making it a broadly applicable marker even in the absence of specific molecular lesions [5]. Importantly, standardized assays for *WT1* quantification have been developed and validated, including recommendations from the ELN regarding cut-off values and assay quality. *WT1* is not AML-specific, but rather reflects abnormal myelopoiesis, and its expression in peripheral blood (PB) has shown superior clinical utility compared to bone marrow due to lower background levels and easier accessibility [6,7].

*WT1* overexpression has been reported across a wide range of AML subtypes and has been associated with specific cytogenetic and molecular features; however, its expression levels and biological significance may vary depending on the underlying disease context, reflecting the intrinsic heterogeneity of AML [8].

Numerous studies have demonstrated the prognostic value of *WT1* monitoring, particularly in patients undergoing allo-HSCT. However, despite promising results, widespread adoption in clinical practice is limited due to a lack of prospective, multicenter studies with sufficient sample size. Some studies have evaluated *WT1* expression in PB before and after transplant, showing correlations with relapse and survival, especially in patients with intermediate-risk AML [9,10,11]. In this prospective multicenter study, we analyzed a cohort of AML patients from Croatia who achieved complete remission and underwent allo-HSCT. The aim of this study was to evaluate the prognostic value of peripheral blood *WT1* expression before and early after transplantation, with a particular focus on its association with relapse risk and survival outcomes.

## 2. Results

### 2.1. Baseline Characteristics

Baseline patient characteristics are summarized in Table 1. A total of 43 patients were included in the study, with a mean age of 48.4 ± 13.8 years; 54% were male. According to the ELN 2022 risk classification, 14% of patients were categorized as favorable, 60% as intermediate, and 26% as adverse-risk. A minority of ELN favorable-risk patients underwent allo-HSCT due to additional high-risk clinical or molecular features identified during the treatment course. *FLT3* mutations were detected in 35% of patients, while *NPM1* mutations were present in 27%. *WT1* expression at diagnosis was available in all patients and was detectable in all evaluable cases. The difference in *FLT3* mutation frequency between Table 1 and Supplementary Table S1 reflects the use of different analysis subsets, as the latter includes only patients with available WT1_30 measurements.

All patients underwent transplantation in complete remission. Induction therapy was intensive in the majority of patients (n = 40; 93%), consisting of standard anthracycline–cytarabine-based regimens (“7 + 3”) in most cases (n = 39) or FLAG (n = 1). The remaining three patients (7%) received azacitidine–venetoclax.

Conditioning regimens were categorized as myeloablative (MAC; n = 28) or reduced-intensity (RIC; n = 15). MAC regimens consisted of FluBu3/4-based or BuCy-based conditioning, whereas RIC regimens consisted of FluBu2-based or FluCyTBI-based conditioning. Graft-versus-host disease (GvHD) prophylaxis was administered according to donor type and institutional standards. These treatment variables were not included in formal analyses due to sample size limitations. Baseline characteristics were broadly comparable between patients with WT1_30 < 3 and ≥3 copies/10^4^ *ABL* (Supplementary Table S1). In addition, transplant-related variables, including donor type, conditioning intensity, graft-versus-host disease, and *FLT3* inhibitor maintenance, were similarly distributed between groups, with no statistically significant differences observed.

Among patients with FLT3-mutated AML, all received midostaurin during induction and consolidation. Post-transplantation, seven patients received maintenance therapy with *FLT3* inhibitors (midostaurin, n = 4; gilteritinib, n = 2; sorafenib, n = 1), while eight did not. Maintenance therapy was typically initiated after day +100.

The median follow-up was 28 months (IQR 22–42). At the last follow-up, 12 patients (28%) had relapsed and 12 (28%) had died. To further evaluate the prognostic value of *WT1* expression, we first performed ROC curve analysis for WT1_pre and WT1_30 in predicting CIR.

### 2.2. ROC Curve Analysis

**Figure 1:**
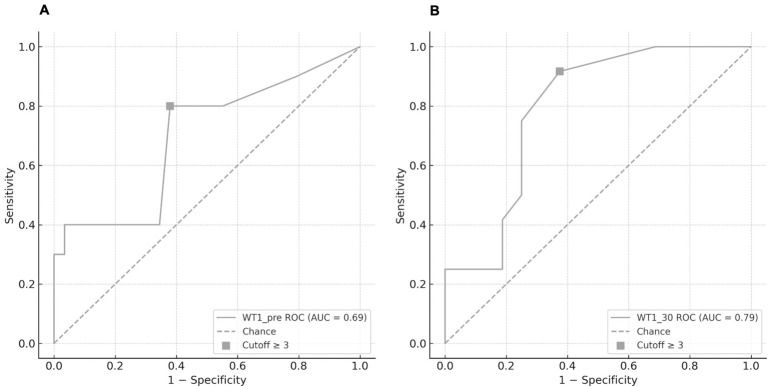
Receiver operating characteristic (ROC) curves for *WT1* expression in predicting cumulative incidence of relapse (CIR) after allo-HSCT. (**A**) WT1_pre (AUC = 0.69, 95% CI 0.47–0.89, *p* = 0.037). (**B**) WT1_30 (AUC = 0.79, 95% CI 0.60–0.95, *p* = 0.005). The square marker indicates the selected cutoff (≥3 copies/10^4^ *ABL*), and the dashed line represents chance performance.

ROC analysis for WT1_30 showed an AUC of 0.79 (95% CI 0.60–0.95, *p* = 0.005). The optimal cutoff value was ≥3 copies/10^4^ ABL, with a sensitivity of 91.7% and a specificity of 62.5%. For WT1_pre, the AUC was 0.69 (95% CI 0.47–0.89, *p* = 0.037), with the same cutoff, yielding a sensitivity of 80.0% and a specificity of 62.1% (Figure 1).

### 2.3. Kaplan–Meier Analysis

**Figure 2:**
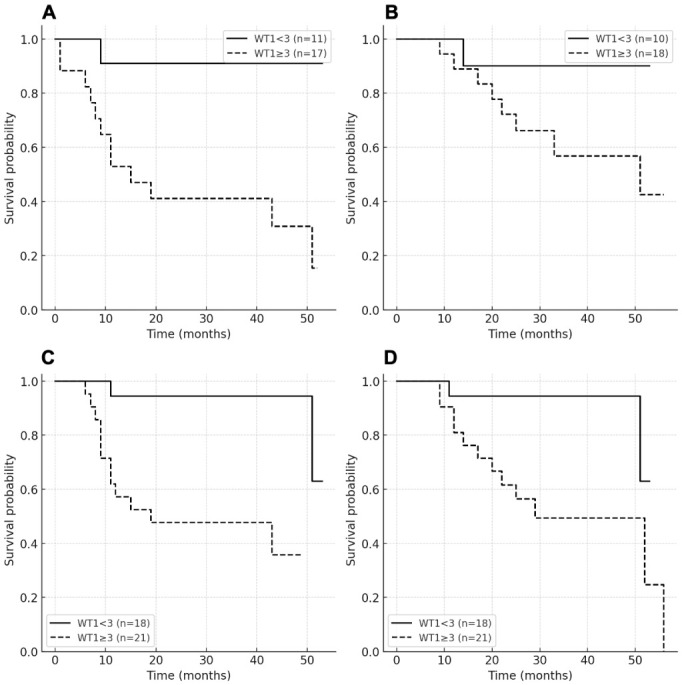
Kaplan–Meier curves for progression-free survival (PFS) and overall survival (OS). (**A**) WT1_30 PFS. (**B**) WT1_30 OS. (**C**) WT1_pre PFS. (**D**) WT1_pre OS. Patients were stratified using a cutoff of ≥3 copies/10^4^ ABL. Analyses were restricted to patients with evaluable *WT1* measurements at the respective time points.

Among patients with evaluable *WT1* expression at day +30 post-transplant (n = 28), progression-free survival (PFS) did not reach the median in patients with WT1_30 < 3, whereas patients with WT1_30 ≥ 3 had a median PFS of 15 months (95% CI 7–51). Importantly, no patients relapsed or died prior to day +30, indicating that missing WT1_30 measurements were unlikely to be related to early clinical outcomes. At 12 months, PFS was 90.9% versus 52.9%, respectively (log-rank *p* = 0.0059). Patients with WT1_30 < 3 had a significantly lower risk of progression compared to those with WT1_30 ≥ 3 (HR 0.21, 95% CI 0.07–0.64). For overall survival (OS), the median was not reached (NR) in the WT1_30 < 3 group, whereas patients with WT1_30 ≥ 3 had a median OS of 51 months (95% CI 22–NR). At 12 months, OS was 90.9% versus 72.2%, respectively (log rank *p* = 0.118). The difference did not reach statistical significance, although the HR suggested a lower risk of death for WT1_30 < 3 (HR = 0.34, 95% CI 0.08–1.32). Among patients with evaluable pre-transplant *WT1* measurements (n = 39), median PFS was NR in the WT1_pre < 3 group, whereas patients with WT1_pre ≥ 3 had a median PFS of 19 months (95% CI 9–NR). At 12 months, PFS was 94.4% versus 57.1%, respectively (log-rank *p* = 0.0008). Patients with WT1_pre < 3 had a significantly lower risk of progression compared to those with WT1_pre ≥ 3 (HR = 0.15, 95% CI 0.05–0.45). For OS, the median was NR in the <3 group, while patients with WT1_pre ≥ 3 had a median OS of 29 months (95% CI 17–NR). At 12 months, OS was 94.4% versus 57.1%, respectively (log-rank *p* = 0.0008). Patients with WT1_pre < 3 had a significantly lower risk of death compared to those with WT1_pre ≥ 3 (HR = 0.23, 95% CI 0.08–0.69) (Figure 2).

### 2.4. Multivariable Cox Analysis

In a multivariable Cox model including ELN 2022 risk category, WT1_30 ≥ 3 copies/10^4^ ABL remained independently associated with inferior PFS (HR 10.5, 95% CI 1.32–78.4, *p* = 0.026), whereas ELN adverse risk was not independently associated with PFS (HR 2.3, 95% CI 0.68–6.88, *p* = 0.188). The overall model was significant (likelihood ratio test *p* = 0.004) (Table 2).

### 2.5. Comparison of WT1 Expression Levels

**Figure 3:**
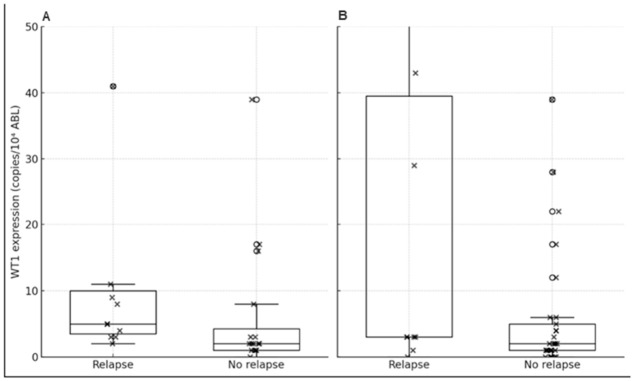
*WT1* expression according to relapse status. (**A**) WT1_30 (median 5.0 [IQR 3.5–10.0] vs. 2.0 [IQR 1.0–4.25], *p* = 0.018). (**B**) WT1_pre (median 3.0 [IQR 3.0–39.5] vs. 2.0 [IQR 1.0–5.0], *p* = 0.069). Data are presented as boxplots with individual data points; horizontal lines indicate medians.

Patients who experienced relapse had significantly higher WT1_30 expression compared to non-relapsed patients (median 5.0 [IQR 3.5–10.0] vs. 2.0 [IQR 1.0–4.25], *p* = 0.018, Mann–Whitney U test) (Figure 3A). For WT1_pre, relapsed patients showed a trend toward higher expression compared to non-relapsed patients (median 3.0 [IQR 3.0–39.5] vs. 2.0 [IQR 1.0–5.0], *p* = 0.069), although this did not reach statistical significance (Figure 3B).

### 2.6. Cumulative Incidence of Relapse (CIR)

**Figure 4:**
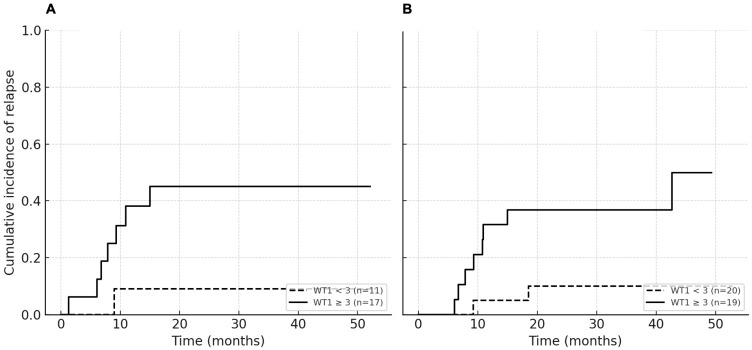
Cumulative incidence of relapse (CIR) according to *WT1* expression. (**A**) WT1_30, stratified by the cutoff ≥ 3 copies/10^4^ ABL. At 12 months, CIR was 31% for WT1_30 ≥ 3 versus 9% for WT1_30 < 3 (Gray’s test *p* = 0.054). (**B**) WT1_pre, stratified by the same cutoff. At 12 months, CIR was 21% for WT1_pre ≥ 3 versus 5% for WT1_pre < 3 (Gray’s test *p* = 0.023).

Of the 12 deaths observed during follow-up, four occurred without prior relapse and were classified as non-relapse mortality. Among patients with evaluable *WT1* expression at day +30 post-transplant (n = 28), at 12 months, the CIR was 31% for patients with WT1_30 ≥ 3 compared with 9% for WT1_30 < 3 (Gray’s test *p* = 0.054). Among patients with evaluable pre-transplant WT1 measurements (n = 39), the 12-month CIR was 21% for WT1_pre ≥ 3 compared with 5% for WT1_pre < 3 (Gray’s test *p* = 0.023; Figure 4). Given the limited number of relapse events and the comparable baseline characteristics between patients with WT1_30 < 3 and ≥3 copies/10^4^ *ABL*, multivariable competing-risk regression for CIR was not performed to avoid model overfitting.

Curves were estimated using the Aalen–Johansen method, treating death without relapse as a competing event. Groups were compared using Gray’s test. Analyses were restricted to patients with evaluable *WT1* measurements at the respective time points.

## 3. Discussion

In this prospective multicenter study, we evaluated *WT1* expression in peripheral blood (PB) as a biomarker of measurable residual disease (MRD) and cumulative incidence of relapse (CIR) in patients with AML undergoing allo-HSCT. Our findings demonstrate that *WT1* expression, both before transplantation and at day +30, provides significant prognostic information. Patients with *WT1* expression ≥ 3 copies/10^4^ *ABL* at either time point had a substantially higher risk of relapse and inferior PFS, with day +30 also showing a trend toward inferior OS; however, this did not reach statistical significance. These findings support the clinical value of *WT1* monitoring in the transplant setting and add to the growing body of evidence that PB testing can serve as a practical MRD tool in AML.

*WT1* encodes a zinc-finger transcription factor that is aberrantly expressed in the majority of AML cases, making it one of the most widely applicable molecular MRD markers [5]. Unlike mutation-specific targets such as *NPM1*, *WT1* monitoring can be performed in nearly all patients, regardless of mutational profile [12]. However, interpretation of *WT1* levels has been challenging because the gene is not AML-specific and can be variably expressed in normal hematopoiesis [5]. Standardization efforts, including those led by the ELN, have improved assay reproducibility and suggested threshold values for clinical use [12].

Our findings align with prior studies demonstrating that *WT1* expression has prognostic value both before and after allo-HSCT. Elevated pre-transplant *WT1* levels have been repeatedly associated with higher relapse risk and shorter survival [10,13,14]. Similarly, several groups have identified day +30 *WT1* expression as a critical determinant of post-transplant outcomes [9,11,15]. Recent studies further support the role of *WT1* as a post-transplant MRD marker, although variability in thresholds and time points remains a significant limitation [16]. Our study confirms these observations in a prospective, multicenter cohort and extends them by demonstrating consistent results across both pre-transplant and day +30 time points. These findings further support the role of *WT1* expression as a marker of disease burden associated with adverse outcomes in AML [8]. However, in contrast to previous reports in which *WT1* did not retain independent prognostic significance in multivariable analyses, WT1_30 in the present study remained an independent predictor of PFS, suggesting that its prognostic value may be particularly relevant in the early post-transplant setting. The observed differences in outcomes are therefore unlikely to be explained by baseline or treatment-related factors alone. Comparisons between WT1_pre and WT1_30 should be interpreted cautiously, as these analyses were performed in partially overlapping patient subsets.

An important consideration in *WT1* monitoring is the optimal cut-off for defining MRD positivity. The ELN and earlier studies have often suggested 50 copies/10^4^ *ABL* in peripheral blood as a practical threshold to minimize false positives [5,12]. In contrast, our ROC analyses identified a lower empirical cut-off of ≥3 copies/10^4^ *ABL* that effectively discriminated between patients with high and low relapse risk. While this discrepancy may appear striking, several points warrant discussion. First, Cilloni and colleagues demonstrated that *WT1* levels in healthy peripheral blood are typically very low, with a median close to 1 copy/10^4^ *ABL* [5]. The higher threshold of 50 copies was therefore proposed primarily as a conservative safeguard against false positives rather than a biologically defined level.

Recent data further support the notion that this conventional threshold may be overly conservative and may limit sensitivity for MRD detection. In a recent study, a lower threshold of 7 copies/10^4^ *ABL* improved sensitivity and concordance with established molecular markers while maintaining acceptable specificity [17]. These findings support the concept that lower *WT1* thresholds may retain clinical relevance, particularly in clinical settings characterized by low disease burden, such as patients undergoing transplantation in complete remission. In this context, the lower cutoff identified in our study may reflect cohort-specific characteristics and a lower baseline disease burden. However, we acknowledge that lower *WT1* thresholds may increase the risk of false-positive results due to physiological *WT1* expression in normal hematopoiesis, and these findings should therefore be interpreted with caution and require external validation.

In our study, *WT1* measurements at later time points were limited and not systematically available, precluding robust analysis of longitudinal *WT1* dynamics. Therefore, our findings primarily support the prognostic value of a single early post-transplant measurement at day +30, while the role of longitudinal *WT1* monitoring requires further investigation.

Notably, WT1_30 remained independently associated with PFS after adjustment for ELN 2022 risk category, suggesting that early post-transplant *WT1* expression may capture residual disease burden beyond baseline genetic risk stratification. This threshold should therefore be considered exploratory. While the ≥3 copies/10^4^ *ABL* threshold performed well in our dataset, validation in larger, independent cohorts will be essential before clinical adoption. Nonetheless, our findings highlight that fixed universal thresholds may not be appropriate across all settings, and that *WT1* interpretation may need to be tailored according to patient selection and clinical context.

*WT1* monitoring also has important practical implications. In routine clinical practice, MRD assessment is typically based on mutation-specific PCR [18] or multiparameter flow cytometry [19], while chimerism analysis is commonly used after allo-HSCT but primarily reflects engraftment dynamics and is less sensitive for relapse prediction [12,20]. Each of these approaches has inherent limitations: mutation-specific assays are applicable only to selected patient subsets, flow cytometry requires significant technical expertise, and chimerism lacks sensitivity for early disease detection. In contrast, *WT1* offers broad applicability and can be measured in peripheral blood, reducing the need for invasive bone marrow sampling.

In our center, post-transplant interventions such as donor lymphocyte infusion, hypomethylating agents, or targeted therapies are typically initiated at the time of overt relapse rather than in a pre-emptive setting. Although a subset of patients received *FLT3* inhibitor maintenance, its distribution was balanced between WT1-defined groups, and early relapses frequently occurred before the typical time point for maintenance initiation, suggesting that maintenance therapy is unlikely to have confounded the observed associations.

Taken together, these findings support *WT1* as a practical adjunct to existing MRD strategies for early post-transplant risk assessment. However, its clinical integration should be guided by standardized protocols and validated thresholds, which will require prospective multicenter validation.

The strengths of our study include its prospective design, multicenter participation, and systematic sampling at predefined time points. To our knowledge, this represents one of the few prospective multicenter cohorts systematically evaluating *WT1* expression in PB before and after allo-HSCT. The availability of parallel molecular and clinical data allowed robust evaluation of the biomarker’s prognostic significance.

Limitations should be acknowledged. The sample size was relatively modest (n = 43), which reduced statistical power, particularly for subgroup analyses. This reflects the prospective, multicenter design of the study and the limited number of eligible patients undergoing allo-HSCT within the study period. Consequently, the statistical power, particularly for multivariable analyses and subgroup comparisons, is limited and the findings should be interpreted with caution. Median follow-up was approximately 28 months, adequate for relapse assessment but not for long-term survival outcomes. Although samples were collected at six predefined time points, only two were analyzed in the present study due to incomplete data availability at later time points, which precluded robust statistical analysis. Therefore, we focused on pre-transplant and day +30 measurements, as these had the highest data completeness and are clinically most relevant for early post-transplant risk stratification. Analysis of additional time points is ongoing and will be addressed in future studies. In multivariable analysis, WT1_30 remained independently associated with PFS after adjustment for ELN risk category, supporting its potential role as an early post-transplant risk stratification marker. However, the wide confidence interval reflects the limited number of events and warrants cautious interpretation. Potential confounding factors were partially addressed by incorporating ELN 2022 risk into the multivariable model; however, residual confounding cannot be excluded given the limited sample size and observational study design. In particular, relevant molecular and treatment-related variables, such as *FLT3* and *NPM1* mutation status, maintenance therapy, and graft-versus-host disease, were not included in the multivariable model due to sample size constraints, and their potential contribution to the observed associations cannot be fully excluded.

Importantly, key transplant-related variables, including donor type, conditioning intensity, and graft-versus-host disease, were balanced between WT1-defined groups, supporting that the observed associations were unlikely to be driven by major clinical confounders. Although *WT1* measurements were not available for all patients at day +30, missing data were primarily related to real-world logistical factors rather than early clinical events, as no patients relapsed or died prior to this time point. Analyses restricted to the WT1_30 subgroup should therefore be interpreted with additional caution due to the reduced sample size and corresponding limitations in statistical power. Furthermore, the WT1_30 analysis was restricted to patients who were alive and relapse-free at day +30, consistent with a landmark analysis approach. This may introduce potential landmark bias by excluding early events, and the findings should therefore be interpreted within this methodological context.

Future research should focus on refining the integration of *WT1* into MRD monitoring algorithms. This includes determining whether dynamic changes in WT1 expression across multiple post-HSCT time points add prognostic value beyond single measurements, and whether combining *WT1* with mutation-specific assays or flow cytometry can enhance predictive accuracy. Moreover, prospective interventional trials are needed to test whether WT1-guided strategies, such as pre-emptive donor lymphocyte infusion or maintenance therapy, can improve clinical outcomes.

## 4. Materials and Methods

This prospective multicenter study included adult AML patients from six hematology centers across Croatia, with all allogeneic transplant procedures performed at the national transplant center (University Hospital Centre Zagreb). Patients were eligible for inclusion if they were candidates for allogeneic hematopoietic stem cell transplantation (allo-HSCT) and met the following criteria: (1) age ≥ 18 years; (2) AML in complete remission (CR/CRi) prior to transplantation; (3) detectable *WT1* expression at diagnosis; and (4) signed informed consent. Exclusion criteria included failure to proceed to allo-HSCT due to disease progression or clinical unfitness, and insufficient follow-up data.

The target sample size of approximately 40 patients was defined based on feasibility and expected case accrual across participating centers. Ultimately, all consecutive eligible patients meeting the inclusion criteria during the study period were included. All patients received induction chemotherapy and achieved complete remission before undergoing allo-HSCT, which was performed at the University Hospital Centre Zagreb, the national referral center for stem cell transplantation. Informed consent was obtained from all participants.

PB samples were collected at six predefined time points: at diagnosis, before transplantation, and at 1, 2, 3, and 6 months after allo-HSCT. For the present analysis, we focused on pre-transplant (WT1_pre) and day +30 (WT1_30) time points due to the highest data completeness and their clinical relevance for early post-transplant risk stratification. WT1_pre analyses were performed in patients with available measurements (n = 39), while WT1_30 analyses were restricted to patients with evaluable samples at day +30 (n = 28). A complete-case approach was therefore used. Analyses involving *WT1* expression at day +30 (WT1_30) were restricted to patients who were alive and relapse-free at that time point and can therefore be interpreted as an early post-transplant landmark analysis. These time points are also the most frequently reported in previous studies. Further analyses of additional time points are planned for subsequent studies. The reduced availability of WT1_30 measurements reflects real-world multicenter logistics and was not related to early relapse or death, as no patients experienced these events before day +30. In addition to *WT1* assessment, molecular characterization at diagnosis included targeted PCR analysis for *NPM1* and *FLT3* (ITD and TKD) mutations. In a subset of patients (n = 15), additional next-generation sequencing (NGS) using a targeted myeloid panel was performed. Selected molecular alterations (e.g., *CEBPA* or *KMT2A*) were assessed in individual cases; however, extended molecular profiling was not systematically available across all centers and was not included in the present analysis. Cytogenetic analysis (karyotype) was available for all patients, enabling standardized ELN 2022 risk stratification.

*WT1* expression was measured in PB using a standardized, commercially available real-time quantitative PCR kit (*WT1* ProfileQuant, IPSOGEN, Marseille, France). The assay was performed according to the manufacturer’s instructions with established quality control procedures. Expression levels were expressed as the number of *WT1* copies per 10^4^ ABL copies. Molecular testing for *FLT3* and *NPM1* mutations was performed using PCR at diagnosis. In a subset of 15 patients, additional mutational profiling was conducted using a targeted myeloid next-generation sequencing panel.

Descriptive statistics were used to summarize baseline and transplant-related patient characteristics, including age, sex, ELN 2022 risk category, response to induction therapy, donor type, conditioning intensity, graft-versus-host disease, maintenance therapy, and molecular features. Categorical variables were reported as frequencies and percentages, and continuous variables were reported as mean ± standard deviation or median (interquartile range), as appropriate based on data distribution. *WT1* expression analysis was performed using receiver operating characteristic (ROC) curves to define optimal cutoffs for predicting relapse at any time during follow-up, with the optimal threshold determined using the Youden index (maximum sensitivity + specificity − 1). Relapse was treated as a binary outcome for ROC analysis. ROC analyses, including calculation of the AUC, 95% confidence intervals (CI), *p*-values, and figure generation, were conducted in Python (version 3.10) using the scikit-learn and matplotlib libraries. Survival outcomes were analyzed using the Kaplan–Meier method, with comparisons between groups performed using the log-rank test. Progression-free survival (PFS) was defined as the time from initiation of AML treatment to relapse or death from any cause. Overall survival (OS) was defined as the time from initiation of AML treatment to death from any cause. Patients without an event were censored at the time of last follow-up. Hazard ratios (HRs) with 95% confidence intervals (CIs) were estimated using univariable and multivariable Cox proportional hazards models. The CIR was estimated using the Aalen–Johansen method, treating death without relapse as a competing event, and compared between groups using Gray’s test (cmprsk package in R, version 4.3). Differences between groups were evaluated using the Mann–Whitney U test for continuous variables. Descriptive statistics, group comparisons, and Kaplan–Meier analyses were performed using GraphPad Prism version 9.0 (GraphPad Software, San Diego, CA, USA). Given the limited number of events, a parsimonious multivariable Cox proportional hazards model including WT1_30 (≥3 vs. <3 copies/10^4^ ABL) and ELN 2022 risk (adverse vs. non-adverse) was constructed to evaluate the independent prognostic value of WT1_30 for PFS. ELN 2022 risk was dichotomized as adverse versus non-adverse to reduce model complexity and avoid overfitting.

## 5. Conclusions

In summary, our prospective multicenter cohort study demonstrates that *WT1* expression in peripheral blood, both before and 30 days after allo-HSCT, is associated with relapse risk in AML patients. Peripheral blood testing offers a simple, reproducible, and minimally invasive approach to MRD monitoring, applicable to the majority of patients. While our analysis suggests that even low *WT1* levels (≥3 copies/10^4^ *ABL*) may have prognostic significance, this threshold requires validation in larger independent cohorts. Together, these findings support *WT1* as a valuable MRD marker and its integration into post-transplant surveillance strategies.

## Figures and Tables

**Table 1:** Baseline characteristics of the study cohort.

Variable	Value
Age (years)	48.4 ± 13.8
Sex	
Male	23 (54%)
Female	20 (46%)
ELN 2022 risk	
Favorable	6 (14%)
Intermediate	26 (60%)
Adverse	11 (26%)
Response to induction therapy	
Achieved complete remission after induction	31 (72%)
Required additional therapy to achieve remission	12 (28%)
Donor type	
Matched sibling donor (MSD)	10 (23%)
Matched unrelated donor (MUD)	27 (63%)
Haploidentical donor	6 (14%)
Conditioning intensity	
Myeloablative (MAC)	28 (65%)
Reduced-intensity (RIC)	15 (35%)
*FLT3* mutation	
Positive	15 (35%)
Negative	27 (65%)
*NPM1* mutation	
Positive	11 (27%)
Negative	29 (73%)
WT1_pre (copies/10^4^ ABL)	2 (1–6)
WT1_30 (copies/10^4^ ABL)	3 (2–11)
Follow-up (months)	28 (22–42)
Relapse	12 (28%)
Dead at last follow-up	12 (28%)

Data are presented as mean ± standard deviation for normally distributed variables, median (IQR) for non-normally distributed variables, and n (%) for categorical variables. *NPM1* mutation status was available for 40 patients. *FLT3* mutation status was available for 42 patients.

**Table 2:** Multivariable Cox proportional hazards model for PFS.

Variable	Hazard Ratio (HR)	95% CI	*p*-Value
WT1_30 ≥ 3 copies/10^4^ ABL	10.5	1.32–78.4	0.026
ELN 2022 adverse risk	2.3	0.68–6.88	0.188

Multivariable Cox model including WT1_30 (≥3 vs. <3 copies/10^4^ ABL) and ELN 2022 risk category (adverse vs. non-adverse). N = 28; number of events = 13. Overall model likelihood ratio test *p* = 0.004.

## Data Availability

The data presented in this study are available upon request from the corresponding author.

## Supplementary Materials

The following supporting information can be downloaded at: https://www.mdpi.com/article/10.3390/ijms27104367/s1.
