# Supplementary material for: Early Peripheral Blood WT1 Expression Predicts Relapse After Allogeneic Hematopoietic Stem Cell Transplantation in Acute Myeloid Leukemia
Source: Int J Mol Sci. 2026 May 14;27(10):4367. doi: 10.3390/ijms27104367 (PMC13207779; doi:10.3390/ijms27104367)
Supplement: Supplementary file 1 [file ijms-27-04367-s001.zip › ijms-4297551-supplementary.pdf]

Table S1. Baseline characteristics stratified by *WT1* expression at day +30.

Variable	WT1_30 <3 (n = 11)	WT1_30 ≥3 (n = 17)	p-value
**Age (years)**	51 (44–60)	46 (31–52)	0.14
**Sex**			0.12
Male	3 (27%)	11 (61%)	
Female	8 (73%)	6 (39%)	
**ELN 2022 risk**			0.87
Favorable	1 (9%)	3 (17%)	
Intermediate	7 (64%)	9 (50%)	
Adverse	3 (27%)	5 (28%)	
***FLT3* mutation**			1.00
Positive	4 (40%)	7 (41%)	
Negative	6 (60%)	10 (59%)	
***NPM1* mutation**			1.00
Positive	3 (30%)	5 (31%)	
Negative	7 (70%)	11 (69%)	
**Donor type**			0.33
MSD	2 (18%)	5 (29%)	
MUD	6 (55%)	11 (65%)	

Haploidentical	3 (27%)	1 (6%)	
**Conditioning intensity**			0.34
MAC	8 (73%)	15 (88%)	
RIC	3 (27%)	2 (12%)	
**GVHD**			1.00
Yes	5 (45%)	8 (47%)	
No	6 (55%)	9 (53%)	
***FLT3* inhibitor maintenance**			0.58
Yes	2 (50%)	2 (29%)	
No	2 (50%)	5 (71%)	

Baseline characteristics are shown for patients with evaluable *WT1* expression at day +30 post-transplant (n = 28). Data are presented as median (interquartile range) or n (%). p-values were calculated using the Mann–Whitney U test for continuous variables and Fisher's exact test for categorical variables, as specified a priori. Due to missing data, totals for some variables may not sum to the group n. GVHD includes both acute and chronic graft-versus-host disease. Analyses for *FLT3* inhibitor maintenance were restricted to FLT3-mutated patients.
